# Hospital admissions due to infectious and parasitic diseases in England and Wales between 1999 and 2019: an ecological study

**DOI:** 10.1186/s12879-022-07388-1

**Published:** 2022-04-23

**Authors:** Kanar Sweiss, Abdallah Y. Naser, Mohammed Samannodi, Hassan Alwafi

**Affiliations:** 1 Department of Basic Pharmaceutical Sciences, Faculty of Pharmacy, Isra University, Amman, Jordan; 2 Department of Applied Pharmaceutical Sciences and Clinical Pharmacy, Faculty of Pharmacy, Isra University, Amman, Jordan; 3 Faculty of Medicine, Umm AlQura University, Mecca, Saudi Arabia

**Keywords:** England, Hospitalization, Infectious, Parasitic, United Kingdom, Wales

## Abstract

**Background:**

Infectious diseases continue to account for considerable illness and death worldwide, and emerging infectious diseases (EIDs) are a significant burden on global economies and public health. This study aimed to investigate the trends in infectious and parasitic disease (IPD) hospital admissions (HA) in England and Wales between 1999 and 2019.

**Methods:**

This is an ecological study using publicly available data taken from the Hospital Episode Statistics database in England and the Patient Episode Database for Wales. Hospital admission data were collected for the period between April 1999 to March 2019. IPDHA were identified using the tenth version of the International Statistical Classification of Diseases system, diagnostic codes (A00–B99). The trend in hospital admissions was assessed using a Poisson model.

**Results:**

The overall annual number for IPD hospital admissions for various causes increased by 412.9%, from 151,336 in 1999 to 776,215 in 2019, representing an increase in hospital admission rate of 349.9% from 290.25 (95% CI 288.79–291.71) in 1999 to 1305.88 (95% CI 1303.00–1308.77) in 2019 per 100,000 persons: trend test, p < 0.01. The most common causes of infectious and parasitic disease hospital admissions were intestinal infectious diseases, other bacterial diseases, and other viral diseases, which accounted for 33.6, 27.5, and 23.8%, respectively. Patients aged 15 years and below accounted for 34.2% of the entire number of IPD hospital admissions, followed by the age group 15–59 years with 27.9%, the age group 75 years and above with 22.7%, and then the age group 60–74 years with 15.2%.

**Conclusion:**

There was an increase in the hospital admission rate due to infectious diseases in the UK from 1999 to 2019. The most common causes of infectious and parasitic disease hospital admissions were intestinal infectious diseases, other bacterial diseases, and other viral diseases.

**Supplementary Information:**

The online version contains supplementary material available at 10.1186/s12879-022-07388-1.

## Background

Infectious diseases continue to account for considerable illness and death worldwide, and emerging infectious diseases (EIDs) have become a significant burden on global economies and public health [1]. Infectious diseases are major burdens on the UK health system and economy, accounting for 7% of deaths and annual costs of £30bn [2].

The emergence of infectious diseases is mainly driven by socio-economic, environmental and ecological factors [3]. Factors such as better hygiene, accessibility to medications and improvement in the health care system, have improved the percentage of patients requiring hospital admission or dying due to infectious diseases over previous centuries [4].

Infectious diseases are one of the major causes that can lead to hospital admission [5]. Although sanitation, immunization and public health have lowered the burden of infectious diseases, they have not reduced the rate of hospitalizations due to infectious diseases [6, 7].

Morbidity and mortality related to infectious diseases have decreased overall in the last decade but are still considered to be high [8]. In 2008, the World Health Organization reported that respiratory infections, diarrheal infections, the human immunodeficiency virus, tuberculosis, and malaria account for 18.3% of all causes of death [9]. Additionally, infectious diseases can be acquired during hospitalization which may impact negatively on the healthcare system and may lead to readmission to hospital. For example, in the United States, between 1998 and 2006, the rate of readmission due to surgical site infection ranged from 1.45% to 6.34% among 525 hospitals [10].

Previous studies in the UK investigating the trends in infectious disease hospitalizations are limited or have been focused on subpopulations or specific infections or organisms but not the overall trend of hospitalization due to infectious diseases. Therefore, this study aimed to investigate the trends of hospital admissions (HA) related to infectious and parasitic disease (IPD) in England and Wales between 1999 and 2019.

## Methods

### Study sources and the population

This was an ecological study using publicly available data extracted from the Hospital Episode Statistics (HES) database in England [11] and the Patient Episode Database for Wales (PEDW) for the period between April 1999 and April 2019 [12]. These two medical databases have been used previously to explore the trends of different health outcomes and associated hospital admissions [13–20]. The HES and PEDW databases contain hospital admission data for patients with infectious and parasitic diseases from all age groups. These are subdivided into four categories: below 15 years (pediatric age group), 15–59 years (middle-age group), 60–74 years (youngest-old age group), and 75 years and above (oldest-old age group). We identified infectious and parasitic diseases (IPDs) leading to hospital admission (HA) using the 10th version of the International Statistical Classification of Diseases (ICD) system. All the hospital admissions related to various types of IPDs in England and Wales were identified by using the ICD diagnostic codes (A00–B99). The HES and PEDW databases record all hospital admissions, outpatient, and accident and emergency (A&E) activities performed at all National Health Service (NHS) trusts, and any independent sector funded by these trusts. Data for hospital admissions in England and Wales are available from the years 1999/2000 onwards. Available data include patient demographics, clinical diagnoses, procedures, and duration of stay. HES and PEDW data are checked regularly to ensure their validity and accuracy [11, 21]. To calculate the yearly hospital admission rate for IPDs, we collected mid-year population data for the period between 1999 and 2019 from the Office for National Statistics (ONS) [22].

### Statistical analysis

Hospital admission rates with 95% confidence intervals (CIs) were calculated using the finished consultant episodes of IPD admissions divided by the mid-year population. The trend in hospital admissions was assessed using a Poisson model. All analyses were conducted using SPSS version 25 (IBM Corp, Armonk, NY, USA).

For the data from Wales, there was no hospital admission for the following ICD codes: A50-A64 and B20-B20. In England, not all age data was available for B20-B20 from 2008/2009 to 2018/2019, for A50–A64 from 2012/2013 to 2018/2019, and A70-A74 from 2011/2012 to 2018/2019. There was no hospital admission data available in England and Wales for B10–B10.

## Results

**Fig. 1:**
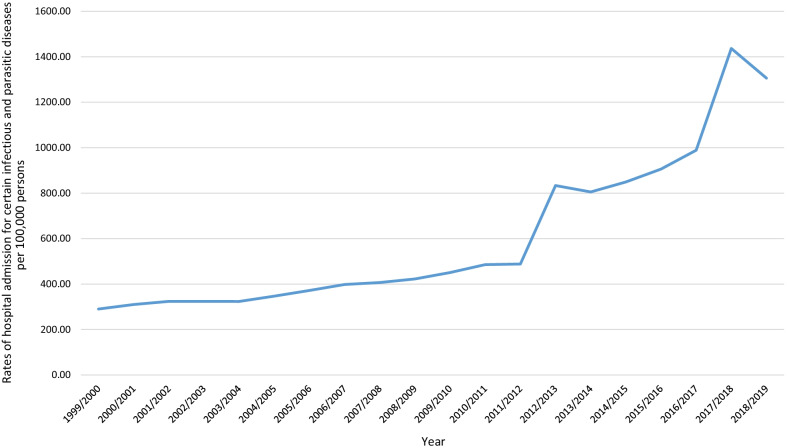
The overall rate of hospital admission related to infectious and parasitic disease during the study period

The overall annual number for IPD hospital admissions for various causes increased by 412.9%, from 151,336 in 1999 to 776,215 in 2019, representing an increase in hospital admission rates of 349.9% from 290.25 (95% CI 288.79–291.71) in 1999 to 1305.88 (95% CI 1303.00–1308.77) in 2019 per 100,000 persons: trend test, p < 0.01, Fig. 1.

The most common causes of infectious and parasitic disease hospital admissions were intestinal infectious diseases, other bacterial diseases, and other viral diseases which accounted for 33.6%, 27.5%, and 23.8%, respectively (Table 1).

**Table 1:** Percentage of IPD admissions from the total number of admissions per ICD code

ICD code	Description	Percentage from total number of admissions (%)
A00-A09	Intestinal infectious diseases	33.6
A15-A19	Tuberculosis	1.9
A20-A28	Certain zoonotic bacterial diseases	0.1
A30-A49	Other bacterial diseases	27.5
A50-A64	Infections with a predominantly sexual mode of transmission	0.8
A65-A69	Other spirochetal diseases	0.1
A70-A74	Other diseases caused by chlamydiae	< 0.1
A75-A79	Rickettsioses	< 0.1
A80-A89	Viral and prion infections of the central nervous system	1.5
A90-A99	Arthropod-borne viral fevers and viral hemorrhagic fevers	< 0.1
B00-B09	Viral infections characterized by skin and mucous membrane lesions	4.9
B10-B10	Other human herpesviruses	–
B15-B19	Viral hepatitis	1.7
B20-B20	Human immunodeficiency virus [HIV] disease	0.7
B25-B34	Other viral diseases	23.8
B35-B49	Mycoses	2.0
B50-B64	Protozoal diseases	0.9
B65-B83	Helminthiases	0.2
B85-B89	Pediculosis, acariasis and other infestations	0.1
B90-B94	Sequelae of infectious and parasitic diseases	< 0.1
B95-B97	Bacterial and viral infectious agents	0.1
B99-B99	Other infectious diseases	0.3

*ICD* International Statistical Classification of Diseases system

Over the past two decades, there has been a significant increase in the rate of hospital admission for other infectious diseases, followed by other bacterial diseases, arthropod-borne viral fevers and viral hemorrhagic fevers, other spirochetal diseases, intestinal infectious diseases, other viral diseases, mycoses, and viral and prion infections of the central nervous system of 18.17, 11.41, 8.77, 4.94, 3.51, 1.94, 1.67, and 1.19-fold, respectively, Table 1. Moreover, the rate of hospital admissions due to certain zoonotic bacterial diseases, helminthiases, viral infections characterized by skin and mucous membrane lesions, rickettsioses, and protozoal diseases have increased by 92.3, 77.3, 51.9, 20.4, and 16.3%, respectively. Detailed information on the rate of hospital admission for each sub-category are available in Additional file 1.

**Fig. 2:**
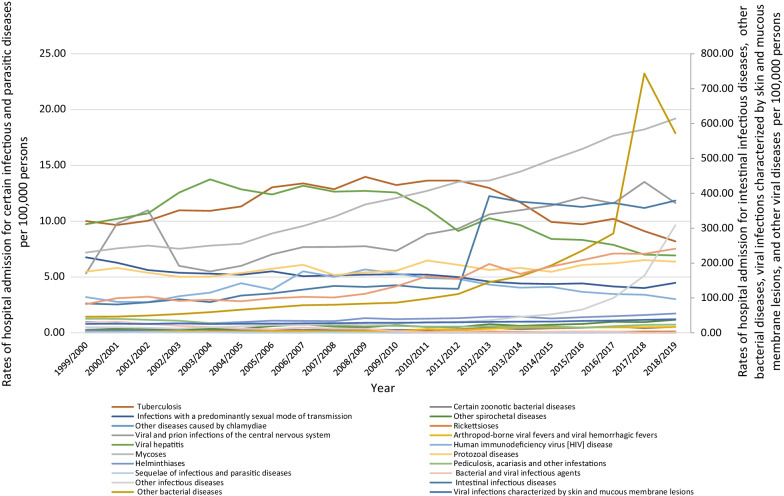
Hospital admission rates due to certain infectious and parasitic diseases in England and Wales stratified by type between 1999 and 2019

However, the rate of hospital admissions due to sequelae of infectious and parasitic diseases, bacterial and viral infectious agents, pediculosis, acariasis and other infestations, other diseases caused by chlamydiae, infections with a predominantly sexual mode of transmission, viral hepatitis, tuberculosis, and human immunodeficiency virus [HIV] disease have decreased by 100.0, 96.8, 42.2, 41.8, 33.7, 28.8, 18.2, and 5.8%, respectively (Fig. 2).

**Fig. 3:**
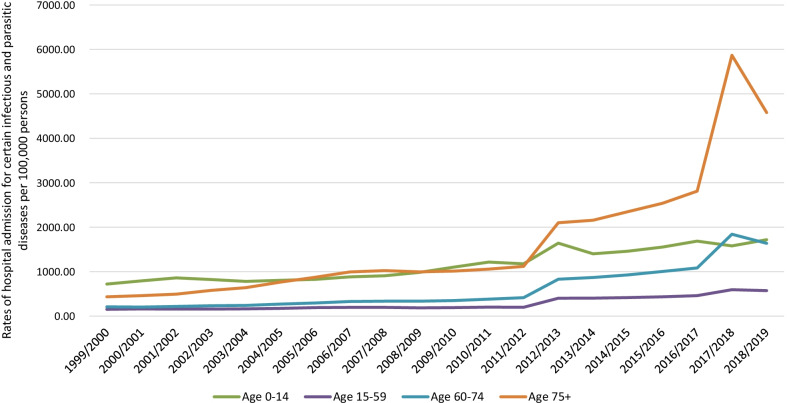
Rates of hospital admission for IPDs in England and Wales stratified by age group

Regarding age group diversity in IPD hospital admissions, the age group below 15 years accounted for 34.2% of the total number of IPD hospital admissions during the study period, followed the age group 15–59 years with 27.9%, the age group 75 years and above with 22.7%, and then the age group 60–74 years with 15.2%. IPD hospital admission rates among patients aged below 15 years increased by 138.3% from 721.08 (95% CI 715.81–726.35) in 1999 to 1718.01 (95% CI 1710.23–1725.79) in 2019 per 100,000 persons, p < 0.01. IPD hospital admission rates among patients aged 15–59 years increased by 276.8% from 152.17 (95% CI 150.81–153.54) in 1999 to 573.32 (95% CI 570.80–575.84) in 2019 per 100,000 persons, p < 0.01. IPD hospital admission rates among patients aged 60–74 years increased by 687.4% from 207.91 (95% CI 204.52–211.30) in 1999 to 1637.04 (95% CI 1628.86–1645.22) in 2019 per 100,000 persons, p < 0.001. IPD hospital admission rates among patients aged 75 years and above increased by 953.9% from 434.56 (95% CI 428.04–441.07) in 1999 to 4579.89 (95% CI 4561.70–4598.07) in 2019 per 100,000 persons, p < 0.001 (Fig. 3).

**Fig. 4:**
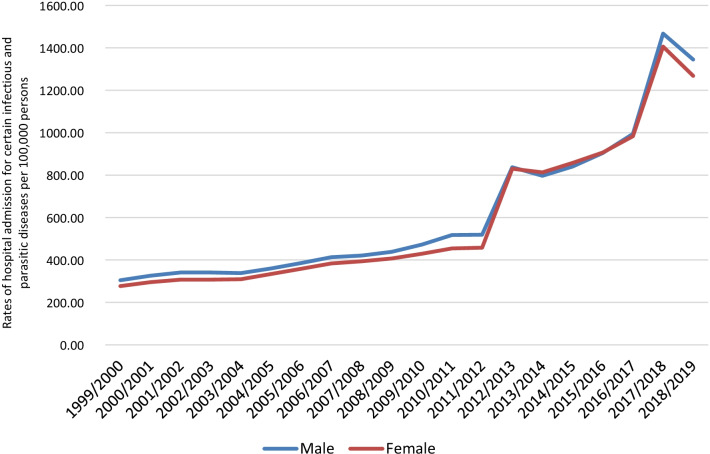
Rates of hospital admission for IPDs in England and Wales stratified by gender

In total, 6,840,714 IPD hospital admission episodes were reported in England and Wales through the study duration. Males contributed to 50.4% of the total number of IPD hospital admissions, accounting for 3,450,850 hospital admission episodes with a mean of 172,542 per year. IPD hospital admission rates among females increased by 358.2% from 276.68 (95% CI 274.69–278.67) in 1999 to 1267.66 (95% CI 1263.66–1271.66) in 2019 per 100,000 persons, p < 0.01. IPD hospital admission rates among males increased by 341.7% from 304.48 (95% CI 302.34–306.62) in 1999 to 1344.90 (95% CI 1340.73–1349.06) in 2019 per 100,000 persons, p < 0.01 (Fig. 4).

### Certain infectious and parasitic disease admission rates by gender

**Fig. 5:**
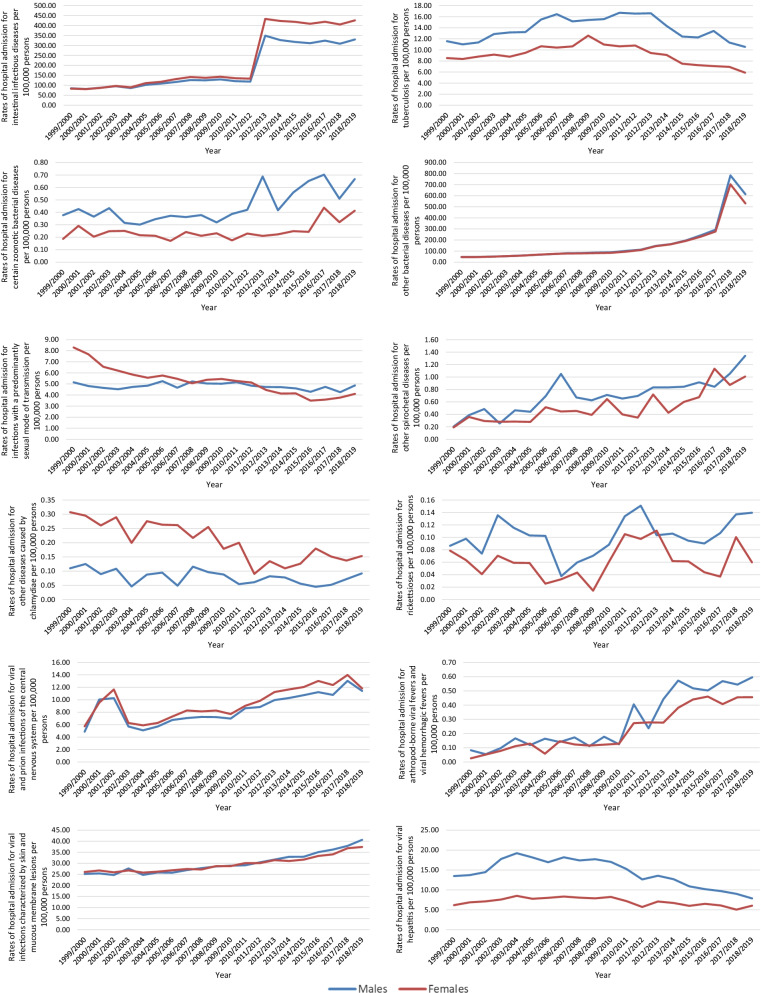

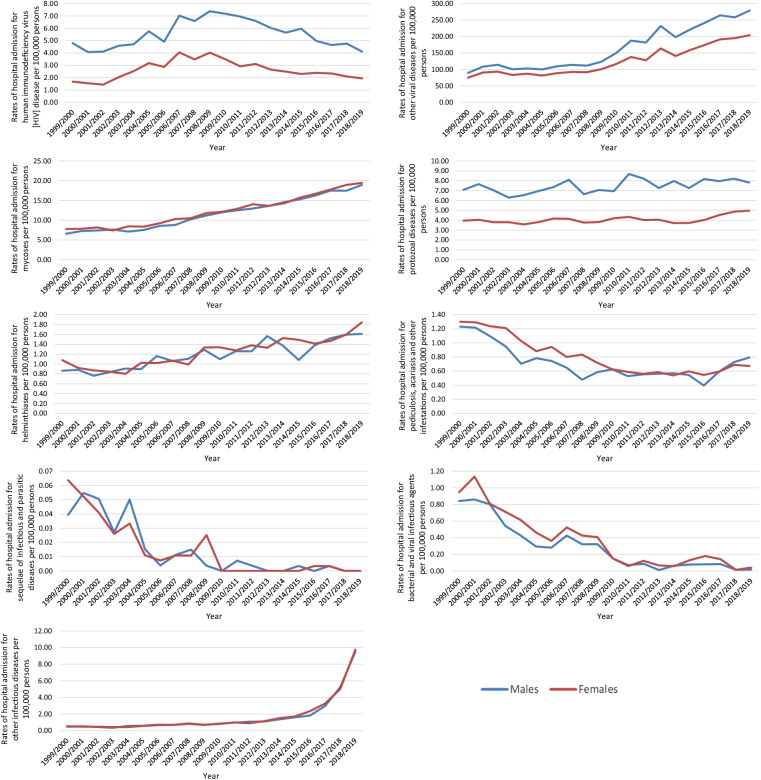
Hospital admission rates for IPDs in England and Wales stratified by gender

IPD hospital admission rates for tuberculosis, certain zoonotic bacterial diseases, other spirochetal diseases, rickettsioses, arthropod-borne viral fevers and viral hemorrhagic fevers, viral hepatitis, human immunodeficiency virus [HIV] disease, protozoal diseases, and other viral diseases were higher among males compared to females, while intestinal infectious diseases, other diseases caused by chlamydiae, and viral and prion infections of the central nervous system were higher among females (Fig. 5).

### Infectious and parasitic diseases admission rate by age group

Hospital admissions due to IPDs for other infectious diseases were observed to be not directly related to age. However, some infections such as rickettsioses, arthropod-borne viral fevers and viral hemorrhagic fevers, viral and prion infections of the central nervous system and viral hepatitis were more common among the younger population (aged 59 years and below).

**Fig. 6:**
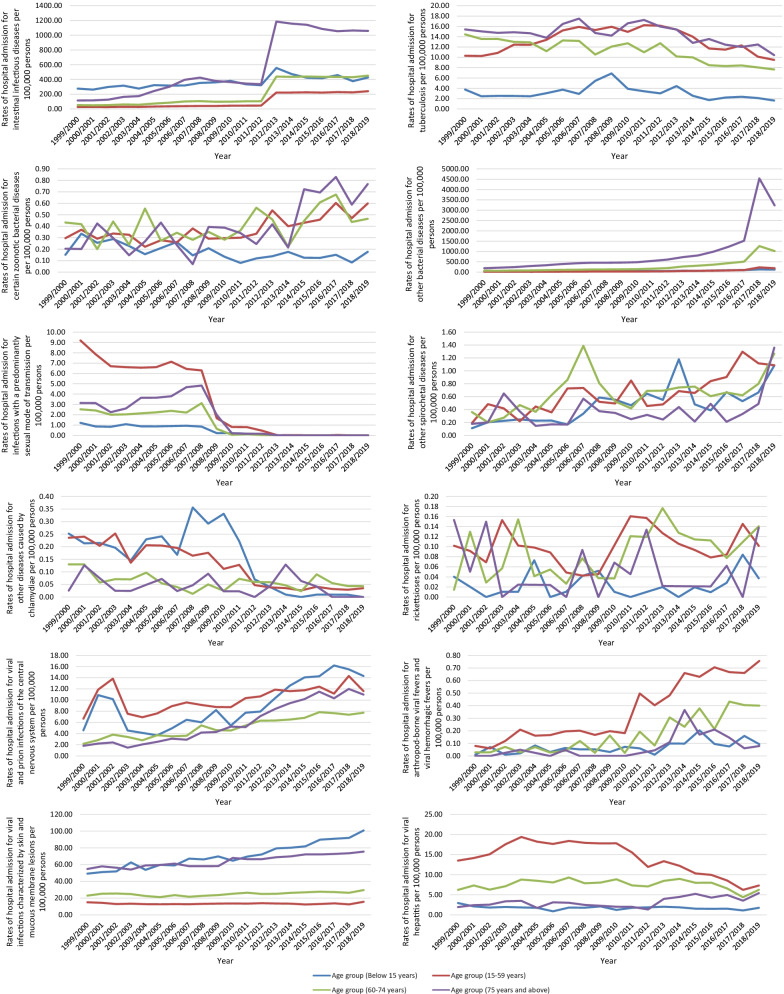

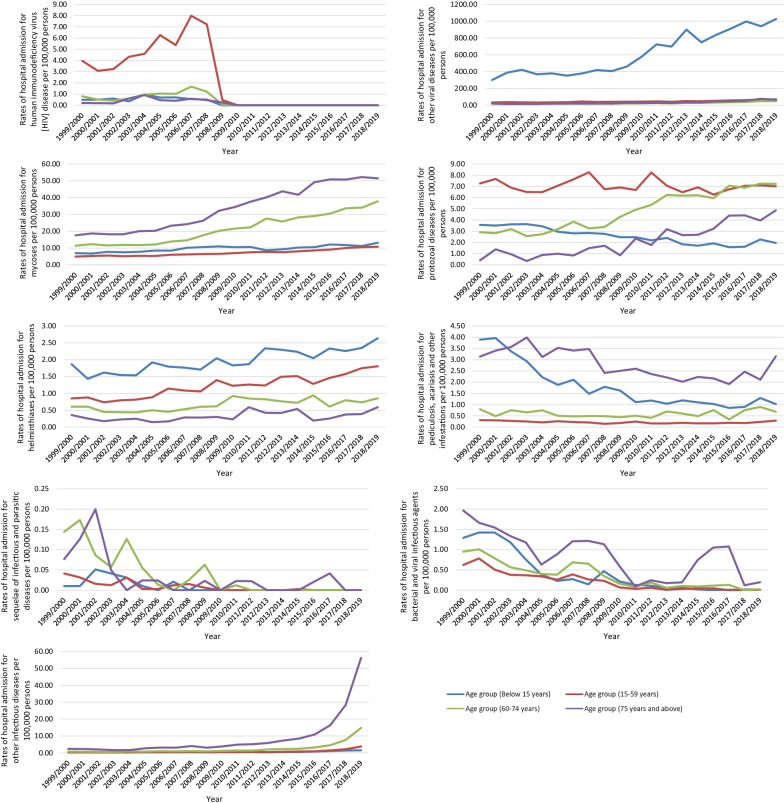
Hospital admission rates for IPDs in England and Wales stratified by age group

Other viral diseases (Cytomegaloviral disease, mumps, infectious mononucleosis, viral conjunctivitis, and viral infection of unspecified site) and helminthiases hospital admission rates were higher among patients aged below 15 years. Infections with a predominantly sexual mode of transmission, arthropod-borne viral fevers and viral hemorrhagic fevers, viral hepatitis, and Human immunodeficiency virus [HIV] disease related hospital admission rates were higher among patients aged 15–59 years. Other bacterial diseases (Leprosy [Hansen's disease], infection due to other mycobacteria, listeriosis, tetanus neonatorum, obstetrical tetanus, other tetanus, diphtheria, whooping cough, scarlet fever, meningococcal infection, streptococcal sepsis, other sepsis, actinomycosis, nocardiosis, bartonellosis, erysipelas, and bacterial infection of unspecified site), mycoses, pediculosis, acariasis and other infestations, bacterial and viral infectious agents, and other infectious diseases related hospital admission rates were higher among patients aged 75 years and above, (Fig. 6).

The most common cause of admission among the age group below 15 years was other viral diseases (cytomegaloviral disease, mumps, infectious mononucleosis, viral conjunctivitis, and viral infection of unspecified site) followed by intestinal infectious diseases which accounted for 53.7% and 31.5% of the total number of hospital admissions for this age group during the study period, respectively, Table 2. The most common cause of admission among the age group 15–59 years was intestinal infectious diseases which accounted for 36.8% of the total number of hospital admissions for this age. Other bacterial diseases (leprosy [Hansen's disease], infection due to other mycobacteria, listeriosis, tetanus neonatorum, obstetrical tetanus, other tetanus, diphtheria, whooping cough, scarlet fever, meningococcal infection, streptococcal sepsis, other sepsis, actinomycosis, nocardiosis, bartonellosis, erysipelas, and cacterial infection of unspecified site) were the most common causes of hospital admissions for the age groups 60–74 years and 75 years and above, which accounted for 48.3 and 56.0% of the total number of hospital admissions for these age groups, respectively, Table 2.

**Table 2:** Percentage of infectious and parasitic disease admissions from the total number of admissions stratified per age group

		Below 15 years		15–59 years		60–74 years		75 years and above	
ICD code	Description	Number of admissions	Percentage from total number of admissions	Number of admissions	Percentage from total number of admissions	Number of admissions	Percentage from total number of admissions	Number of admissions	Percentage from total number of admissions
A00–A09	Intestinal infectious diseases	727,303	31.5%	691,780	36.8%	351,513	34.4%	517,750	33.9%
A15–A19	Tuberculosis	6228	0.3%	87,429	4.6%	17,686	1.7%	12,667	0.8%
A20–A28	Certain zoonotic bacterial diseases	352	< 0.1%	2494	0.1%	652	0.1%	357	< 0.1%
A30–A49	Other bacterial diseases (Leprosy [Hansen's disease], Infection due to other mycobacteria, Listeriosis, Tetanus neonatorum, Obstetrical tetanus, Other tetanus, Diphtheria, Whooping cough, Scarlet fever, Meningococcal infection, Streptococcal sepsis, Other sepsis, Actinomycosis, Nocardiosis, Bartonellosis, Erysipelas, and Bacterial infection of unspecified site)	134,567	5.8%	381,722	20.3%	494,431	48.3%	853,721	56.0%
A50–A64	Infections with a predominantly sexual mode of transmission	882	< 0.1%	21,662	1.2%	1,587	0.2%	1411	0.1%
A65–A69	Other spirochetal diseases	963	< 0.1%	4340	0.2%	1070	0.1%	339	< 0.1%
A70–A74	Other diseases caused by chlamydiae	292	< 0.1%	827	< 0.1%	96	< 0.1%	38	< 0.1%
A75–A79	Rickettsioses	48	0.1%	664	< 0.1%	144	< 0.1%	46	< 0.1%
A80–A89	Viral and prion infections of the central nervous system	18,418	0.8%	68,273	3.6%	8466	0.8%	5,391	0.4%
A90–A99	Arthropod-borne viral fevers and viral hemorrhagic fevers	141	< 0.1%	2428	0.1%	281	< 0.1%	63	< 0.1%
B00–B09	Viral infections characterized by skin and mucous membrane lesions	141,575	6.1%	89,034	4.7%	40,167	3.9%	56,777	3.7%
B10–B10	Other human herpesviruses	0	0	0	0	0	0	0	0
B15–B19	Viral hepatitis	3509	0.2%	93,118	5.0%	12,041	1.2%	2848	0.2%
B20–B20	Human immunodeficiency virus [HIV] disease	522	< 0.1%	15,056	0.8%	607	0.1%	177	< 0.1%
B25–B34	Other viral diseases (Cytomegaloviral disease, Mumps, Infectious mononucleosis, Viral conjunctivitis, and Viral infection of unspecified site)	1,237,328	53.7%	311,282	16.5%	43,842	4.3%	30,255	2.0%
B35–B49	Mycoses	19,416	0.8%	48,018	2.6%	35,529	3.5%	30,293	2.0%
B50–B64	Protozoal diseases	5103	0.2%	46,684	2.5%	7878	0.8%	1,994	0.1%
B65–B83	Helminthiases	3948	0.2%	8087	0.4%	1077	0.1%	287	< 0.1%
B85–B89	Pediculosis, acariasis and other infestations	3550	0.2%	1420	0.1%	956	0.1%	2423	0.2%
B90–B94	Sequelae of infectious and parasitic diseases	17	< 0.1%	56	< 0.1%	54	< 0.1%	26	< 0.1%
B95–B97	Bacterial and viral infectious agents	803	< 0.1%	1439	0.1%	536	0.1%	733	< 0.1%
B99–B99	Other infectious diseases	1231	0.1%	5046	0.3%	4253	0.4%	8265	0.5%

*ICD* International Statistical Classification of Diseases system

## Discussion

This study describes the epidemiology and trends of IPD hospitalizations in the United Kingdom during 1999–2019. The annual rate of hospitalization due to IPDs rose and was consistent with most previous studies [7, 23]. In this study, we found an increase in the rate of hospital admissions among patients aged 75 years and more and patients younger than 15 years old between 1999 and 2019. The most common infectious cause of hospitalization among the whole study population in our study was intestinal infections. In a similar study done in the United States, lower respiratory tract infection was the most common cause of infectious disease hospitalization [10].

In our study, other bacterial diseases (leprosy [Hansen's disease], infection due to other mycobacteria, listeriosis, tetanus neonatorum, obstetrical tetanus, other tetanus, diphtheria, whooping cough, scarlet fever, meningococcal infection, streptococcal sepsis, other sepsis, actinomycosis, nocardiosis, bartonellosis, erysipelas, and cacterial infection of unspecified site) were the most common causes of hospital admissions for the age groups 60–74 and 75 years and above, which accounted for 48.3 and 56.0% of the total number of hospital admissions for these age groups, respectively. Multiple reasons may contribute to the increase in the hospitalization rate due to infectious diseases; one reason may be that the aging population in the UK has increased over the last decades [24]. In addition, older patients are at higher risk of complications, comorbidities, and immunocompromised conditions. These are all risk factors for hospitalization due to infectious diseases [25–27].

In this study, we found that intestinal infections were the most common cause of such hospital admissions. Our results were also consistent with a previous study in the United States, where the authors used a national (nationwide) inpatient sample and reported in their study that GI infections were among the most common causes of hospitalization [28]. In our study, intestinal infectious diseases accounted for 31.5% of the total number of hospital admissions for the age group below 15 years during the study period. We believe that one major reason for the evolution of intestinal infections has been the lack of immunization. For example, rotavirus is the most common cause of infectious gastroenteritis. However, the rotavirus vaccine was only introduced into the UK vaccination schedule in July 2013, which may account for the trend seen in our study [29, 30]. A reasonable goal for a rotavirus vaccine is to replicate the level of illness protection that occurs after natural infection [31]. As a result, the vaccine program's goals include the prevention of moderate to severe rotavirus disease, but not necessarily mild illness [31]. In addition to reducing the number of children admitted to hospitals with dehydration or seen in emergency rooms, an efficient rotavirus vaccination should also reduce the burden on primary care practitioners by reducing the number of office visits or phone calls related to rotavirus gastroenteritis [31]. Finally, in resource-poor nations where rotavirus mortality is high, effective rotavirus vaccines are particularly needed.

Although we found almost equal distribution between males and females in our study, clear differences were noted between gender and rate of hospitalizations, as seen in Fig. 5. These findings are similar to the current literature [10, 32]. In our study, female patients had a higher hospitalization rate than male patients, specifically concerning sexually transmitted diseases such as chlamydia. On the other hand, hospitalization due to digestive system infectious diseases was predominantly linked to male patients.

In this study, we found an increase in the rate of hospital admissions among patients aged 75 years and more and patients younger than 15 years old. These results were also in line with previous studies in the United States [28, 33].

We found that the trend of parasitic and protozoal infections was generally stable throughout the study period. However, for other diseases, such as viral infections, the trend of hospitalization was increasing. A possible explanation for this finding is that better hygiene and public health interventions have been imposed in the community which may influence the trend regarding parasitic and protozoal infections [34], while viral infections reflect the growth of the world population driving the high levels of contagion [27].

Immunization programs are a key public health initiative for the purpose of controlling or reducing the trend of infectious diseases. This was noticed in our study, with many of the viral and bacterial infections being stable over the study period, reflecting good adherence to immunization guidelines, better health care and greater immunization benefits [35].

The prevalence of infectious diseases and their related complications could be minimized through multiple approaches, such as promoting good hygiene hand practice, practicing good safety techniques, adherence to immunization guidelines, and following safer sex guidelines and precautions [36–38].

Our study has several strong points. It is the first study to report the rate of IPD hospitalizations by age and sex in the United Kingdom. Second, the study population was large and representable of the UK population and included a wide variety of infectious disease. Despite the strengths, there were also some limitations. First, it is an ecological study using publicly available databases, and any missing data could limit the conclusions drawn from the study. Additionally, the study was conducted in the United Kingdom, and the findings need validation from studies in other countries. We relied on the international classification of disease (ICD)-9 diagnosis code, which may have poor reliability in identifying the actual diagnosis [32]. The identified temporal variability could be related to potentially non-medical causes, such as updated coding guidelines. These artificial changes may introduce temporal correlations between diagnoses inferred from routine data, violating the assumptions of frequently used statistical methods [39]. Admission data includes admission and readmission at the same time. Therefore, our admission rates could have been overestimated.

## Conclusion

There was an increase in hospital admission rate due to infectious diseases in the UK from 1999 to 2019. The most common infectious and parasitic diseases requiring hospital admission were intestinal infectious diseases, other bacterial diseases, and other viral diseases.

## Supplementary Information

**Additional file 1: Figure S1.** Rates of hospital admission for intestinal infectious diseases per 100,000 persons. **Figure S2.** Rates of hospital admission for viral infections characterized by skin and mucous membrane lesions per 100,000 persons. **Figure S3.** Rates of hospital admission for tuberculosis per 100,000 persons. **Figure S4.** Rates of hospital admission for viral hepatitis per 100,000 persons. **Figure S5.** Rates of hospital admission for other viral diseases per 100,000 persons. **Figure S6.** Rates of hospital admission for viral and prion infections of the central nervous system per 100,000 persons. **Figure S7.** Rates of hospital admission for other bacterial diseases per 100,000 persons.

## Data Availability

Publicly available datasets were analyzed in this study. This data can be found here: http://content.digital.nhs.uk/hes, http://www.infoandstats.wales.nhs.uk/page.cfm?pid=41010&orgid=869.

## Abbreviations

EIDs: Emerging infectious diseases
IPD: Infectious and parasitic disease
HA: Hospital admissions
UK: United Kingdom
HES: Hospital episode statistics
PEDW: Patient episode statistics for Wales
SPSS: Statistical package for social sciences
ICD: International classification of diseases
CI: Confidence interval
