# Supplementary material for: Hospital admissions due to infectious and parasitic diseases in England and Wales between 1999 and 2019: an ecological study
Source: BMC Infect Dis. 2022 Apr 23;22:398. doi: 10.1186/s12879-022-07388-1 (PMC9034500; doi:10.1186/s12879-022-07388-1)
Supplement: Supplementary file 1 — Additional file 1: Figure S1. Rates of hospital admission for intestinal infectious diseases per 100,000 persons. Figure S2. Rates of hospital admission for viral infections characterized by skin and mucous membrane lesions per 100,000 persons. Figure S3. Rates of hospital admission for tuberculosis per 100,000 persons. Figure S4. Rates of hospital admission for viral hepatitis per 100,000 persons. Figure S5. Rates of hospital admission for other viral diseases per 100,000 persons. Figure S6. Rates of hospital admission for viral and prion infections of the central nervous system per 100,000 persons. Figure S7. Rates of hospital admission for other bacterial diseases per 100,000 persons. [file 12879_2022_7388_MOESM1_ESM.docx]

**Supplementary file:**

Figure S1: Rates of hospital admission for intestinal infectious diseases per 100,000 persons

Figure S2: Rates of hospital admission for viral infections characterized by skin and mucous membrane lesions per 100,000 persons

Figure S3: Rates of hospital admission for tuberculosis per 100,000 persons

Figure S4: Rates of hospital admission for viral hepatitis per 100,000 persons

Figure S5: Rates of hospital admission for other viral diseases per 100,000 persons

Figure S6: Rates of hospital admission for viral and prion infections of the central nervous system per 100,000 persons

Figure S7: Rates of hospital admission for other bacterial diseases per 100,000 persons
